# Scientific Items

**Published:** 1880-05-20

**Authors:** 


### SCIENTIFIC ITEMS.

**The Audiphone and Dentaphone.**—That these instruments are of great value in a considerable proportion of cases of deafness there is no reason to doubt, but there is no just ground for the public belief that by their aid the deaf are enabled to hear as well as those with ordinary hearing. On the contrary, they supply, I am convinced, but a very small fraction of normal hearing—much less than a hundredth part. * * * * The simplest osteophone, one that excels by many times the volume of sound transmitted by either audiphone or dentaphone, consists simply of a small rod of hard wood—a convenient size being about two feet long and a quarter of an inch thick—one end of which is placed against the teeth of the speaker, the other resting against or between the teeth of the person hard of hearing. If the speaker now articulates in a natural tone of voice, the vocal vibrations will be transmitted in great volume through the teeth and thence to the ears of the deaf person. Later observations show that it will also convey the voice distinctly when placed against the forehead or other portion of the skull of the hearer. It will also convey perfectly audible speech from the skull of one to that of the other, or in its absence such sounds may be conveyed by simply bringing the heads themselves in contact. Again, instead of the speaker holding it against his teeth, he may place it against the upper part of his chest, when, upon using his voice, the sound will be conveyed as before—of course, independently of the teeth of either person.—DR. THOMAS *in Pacific Medical Journal.*

**Capital Punishment by Electricity.**—We have already referred in the Journal to the proposition that electricity be employed as a means of capital punishment. The plan has been advocated both in France and Germany. A recent writer in the latter country thus sketches the method of procedure: "In a dark room, draped with black, and which is lighted by a single torch—the chamber of execution—there shall stand an iron figure of Justice, with her scales and sword. This goddess will carry a powerful electric battery in her inside; and this battery will be connected with an arm-chair—the seat of death. In front of the chair shall stand the judge's tribunal, and only the judge, jury and other officials shall be present with the condemned during the ceremony of execution. This will consist in the judge reading the story of the crime committed by the prisoner, who will be rigidly manacled to aforesaid chair; and when this has been done the judge will break his rod of office and toss it into one of the scale-pans of the figure of Justice, at the same time extinguishing the solitary torch. The descent of the pan will complete the electric circuit, and shock the wretch into the next world."—*Journal of Chemistry.*

**Power of Imagination.**—Dr. Carpenter says: "A strong direction of the inward consciousness to any part, especially if attended with an expectation of something about to happen, is quite sufficient to change the physical action of a part."

**The Topophone.**—A new instrument, called the *topophone* (from two Greek words meaning *place* and *sound*), has been experimented upon and described by Professor Morton in his report to the Lighthouse Board of the United States, by which the exact direction of sound given by fog-horns or fog-bells may be ascertained. The machinery is very simple, and consists of a vertical rod passing through the roof of the deck cabin, to the upper end of which are attached two adjustable resonators. Below these is a pointer, set at right angles to the bar, while rubber tubes pass through the cabin roof and are connected with a pair of ear-tubes. The whole apparatus can be turned in any direction, and the result is that any person sitting in the cabin, by turning it round until the least sound was percepitible, would bring the pointer within ten degrees, or less than one point, of the direction. The experiments proved that it was easy to ascertain whence the sound came at a distance of from four to six miles.—*Jour. of Chemistry.*

**A Mechanical Watch-Dog.**—Most persons are aware that, by introducing a flame of gas into an open tube, whether of metal or of glass, the tube will sound; in fact, we might easily produce singing flames. The sound, of course, differs according to the size of the tube, the force of the flame, etc. Sometimes the sound is like a roar, at others like a low moan; sometimes high, sometimes low; in fact, the greatest variety of expressions can be produced, according to circumstances. But better than this has been found. There are silent speaking tubes; that is to say, tubes that under ordinary circumstances do not utter a sound; but, if a door be opened, a draught is created, then the glass vibrates, and the most startling noises result. A glass of this description has been contrived in which, when a jet of gas burns, the sound of a dog barking is produced should the street door be opened. Thus may the house be guarded by a mechanical watch-dog!—*Journal of Chemistry.*

**The Winter of 1879-'80.**—The Pacific Medical Journal remarks: "The conjunction of the planets or some other cause has produced a winter of extraordinary and persistent cold on the Pacific coast of North America and on the Atlantic side of northern Europe, whilst the opposite condition of climate has prevailed in the Atlantic States, showing, if the weather be controlled by planetary or stellar influence, that the same breath may 'blow hot and cold.' An extraordinary death-rate has resulted in the large cities of Europe, the mortality in London rising as high as a ratio of 48 per thousand, and in Paris 42 per thousand. On the Pacific coast no excessive mortality has existed. Persistent and dense fogs have characterized the climate in England, but no such condition has prevailed in California."

**The Metric System.**—The Scientific American says: "A five-cent nickel measures in diameter two centimeters, and weighs five grammes. Five of them placed in a row measure a decimeter, and two weigh a dekagramme. As a kiloliter is a cubic meter, the key to the measure of length is the key to the measure of capacity. Any person, therefore, who is fortunate enough to own a five cent nickel may carry in his pocket the entire metric system of weights and measures."

